# Special Issue “Perspectives and Challenges in Sports Medicine for Combat Sports”

**DOI:** 10.3390/jfmk11020234

**Published:** 2026-06-09

**Authors:** Robert Trybulski

**Affiliations:** 1 Provita Medical Center, 44-240 Żory, Poland; roberttrybulski@proton.me; 2 Faculty of Medicine, Katowice Business University, 40-659 Katowice, Poland

## 1. Introduction

Combat sports occupy a distinctive position within sports medicine [1,2,3]. Combat sports do not only comprise a single sport but a family of weight-category, contact, and collision disciplines in which performance depends on technical skill, tactical decision-making, repeated high-intensity actions, deliberate opponent interaction, and the capacity to recover under dense training and competition schedules [4,5]. Consequently, the medical questions raised by boxing, mixed martial arts (MMA), wrestling, judo, karate, taekwondo, kickboxing, and related disciplines extend beyond acute traumatic injuries [6,7,8,9,10]. These medical issues also include cumulative tissue loading, head impact exposure, rapid weight loss, sleep and recovery disruption, psychological strain, and the emergency recognition of uncommon but potentially severe complications [11,12,13,14].

Since the mid-2010s, the field of combat sports medicine has moved from simply documenting that combat sports are associated with injuries toward a more nuanced appreciation of how injury risk is generated, detected, and managed. Injury definitions and surveillance methods are increasingly being standardized, concussion management has become more structured, and weight-making practices are now viewed as a health and governance issue rather than as an isolated nutritional tactic [12,15,16]. Nevertheless, substantial gaps remain. Training injuries are often under-reported compared with competition injuries; exposure denominators differ between studies; women, youth, and recreational athletes remain under-represented; and the evidence supporting recovery, rehabilitation, and implementation strategies is less mature than evidence describing injury patterns.

This Special Issue, “Perspectives and Challenges in Sports Medicine for Combat Sports”, contributes to this evolution of the field with five complementary articles. Together, these studies address musculoskeletal injury surveillance in karate; body composition assessment in MMA; post-exercise recovery interventions; the effects of weight-cutting practices on sleep, recovery, and injury; and a rare but serious lower limb emergency following repetitive strikes in kickboxing [17,18,19,20,21]. These contributions highlight an important direction in the field: combat sport medicine must integrate epidemiology, monitoring, intervention trials, policy-relevant synthesis, and frontline clinical vigilance.

## 2. Contributions to This Special Issue

The cross-sectional survey by de Wet and Yelverton [17] provides needed data from South African Shotokan karate, a context that had been insufficiently examined in the national combat sports literature. The authors noted a high self-reported injury burden, with knee injuries and muscle strains prominently featuring. The findings highlight a clinically important observation that a meaningful proportion of karatekas continued training despite injury. This finding reframes injury prevention as a dojo-based and training culture issue, not only a competition-medicine problem. The study is considered hypothesis-generating because of its cross-sectional design, self-reported data, and retrospective recall limit causal inference. The value of the study is therefore twofold: providing local baseline evidence and establishing a case for prospective, medically verified, and exposure-normalized surveillance using harmonized reporting standards [13,22].

Muracki et al. [18] approached combat sport medicine from an athlete-monitoring perspective by comparing the body composition and phase angle between MMA and powerlifting athletes. The absence of major between-group differences is informative, cautioning against interpreting anthropometry in isolation. MMA performance and health are shaped not only by lean mass, fat mass, and phase angle but also by the interaction among body composition, training load, technical demands, weight class strategy, hydration status, sleep, injury history, and recovery capacity. This contribution supports a move from static profiling toward repeated, contextualized monitoring. Such an approach is compatible with the wider evidence on MMA athlete profiles and bioelectrical impedance analyses. The study also underlines the need for longitudinal designs that determine whether body composition markers predict performance, injury, illness, or maladaptive weight cycling [23,24].

The randomized controlled trial by Trybulski et al. [19] progresses from description to intervention. In trained MMA athletes exposed to a fatigue protocol, sports massage, and a combined blood flow restriction/cooling intervention improved several post-exercise recovery outcomes compared with those achieved with passive rest. The study is practically relevant because recovery is frequently discussed in combat sports but less often tested under controlled conditions with sport-specific participants. The authors also illustrate the next methodological challenge. Recovery interventions should be evaluated with larger samples, longer follow-ups, active comparators, dose-response designs, and mechanistic markers, acknowledging that athlete response is likely individualized. The implementation of these interventions in combat sports also requires considering feasibility, timing, athlete preference, and interaction with the wider training microcycle [25,26].

In their scoping review, Kużdżał et al. [20] synthesize the evidence on the effects of weight-cutting practices on sleep, recovery, and injury outcomes. The review positions rapid weight loss not merely as a nutrition or body mass tactic but as a sports medicine exposure that may influence tissue stress, fatigue, recovery quality, and injury risk. The findings align with broader warnings that aggressive dehydration and short-term body-mass reduction can impose physiological costs. The methodological heterogeneity of the included literature shows why standardized protocols, clearer time windows, sex-specific analyses, and longitudinal follow-up are required in future studies [27,28].

The case report by Beca et al. [21] adds a clinically distinctive and highly relevant dimension to this Special Issue. Acute compartment syndrome following repeated calf kicks in an elite-level kickboxing athlete illustrates that combat sport injuries are not limited to common contusions, sprains, or lacerations. Rare complications may arise from sport-specific mechanisms, and timely recognition can be limb-saving. The case emphasizes the importance of educating athletes, coaches, event medical teams, and emergency clinicians about red flags such as rapidly progressive pain, tense swelling, pain with passive stretching, paresthesia, and functional deterioration. The report links everyday combat sport exposure to emergency medicine and reminds those in the field that prevention also includes rapid escalation pathways [29]. A summary of the studies included in this Special Issue is provided in Table 1.

## 3. Integrating the Evidence: From Isolated Findings to Systems of Care

A common message from the five articles is that combat sport medicine should be conceptualized as a system rather than a set of isolated clinical encounters. Injury surveillance identifies where and how athletes are harmed. Body composition and phase angle assessments may help characterize readiness and adaptation. Additionally, recovery trial test strategies may preserve function between training sessions. The review synthesizing weight-cutting clarifies harmful practices and evidence gaps, and case reports increase the clinical awareness of rare presentations that can be missed when clinicians expect only common injuries.

This systems perspective is particularly important because the injury risk factors in combat sports often interact. The recovery and training quality of an athlete who repeatedly cuts weight may be impaired, and the athlete may experience poor sleep and altered tissue resilience. An athlete who continues training while injured may accumulate compensatory movement patterns, which increases their exposure to severe injury. A fighter who relies on repeated lower leg strikes or absorbs them in sparring may develop soft tissue damage that initially resembles a routine contusion but evolves into a surgical emergency. These examples show why a narrow model of combat sport medicine based only on diagnosis and return-to-play is insufficient. Combat sport medicine requires surveillance, prevention, monitoring, athlete education, coaching collaboration, evidence-based recovery, and clear thresholds for referral.

## 4. Future Research Priorities

Combat sport injury research should continue to adopt shared definitions, severity categories, and exposure denominators. Prospective surveillance should capture both training and competition; include medical verification where feasible; and report athlete sex, age, competitive level, rule set, protective equipment, weight class, and mechanism of injury. The South African Shotokan karate survey shows the value of local baseline evidence, but future studies should determine incidence, recurrence, time loss, clinical diagnosis, and modifiable risk factors [17].

Athlete monitoring needs to involve longitudinal integration. Body composition, phase angle, hydration status, sleep, training load, neuromuscular performance, and subjective wellness should be interpreted together rather than as isolated measurements. This is especially relevant in weight category sports, where measurements recorded during normal training, weight-cutting, and post-weigh-in refueling may differ in meaning. Future studies should test whether monitoring variables can guide safer weight management, prevent injury, or optimize return-to-performance decisions [18,23,28].

Recovery interventions require more pragmatic and mechanistic research in combat-sport settings. The randomized control trial included in this Special Issue supports the potential value of massage and blood flow restriction/cooling strategies, but larger multicenter trials are needed to compare active recovery modalities, define optimal timing and dosage, assess individual response, and evaluate outcomes that matter to athletes and coaches, including subsequent training quality, sparring performance, soreness, pain, sleep, and readiness [19,25,26,30].

Weight-cutting research should progress beyond documenting prevalence toward testing harm reduction policies and educational strategies. Researchers should examine the effects of weigh-in timing, hydration testing, maximum permissible weight regain, nutritional counseling, and coach education. Particular attention should focus on adolescents, women athletes, repeated seasonal weight cycling, and recreational competitors, because these groups may differ in their physiological and psychosocial vulnerabilities [20,27,31].

Combat sport clinicians should study rare but severe complications using high-quality case reports, registries, and clinical pathways. The report of acute compartment syndrome after repeated calf kicks illustrates how sport-specific mechanisms can create atypical emergencies [29]. Researchers should develop educational materials for gyms, event medical teams, and emergency departments, emphasizing red flag symptoms, time-sensitive referral, and the distinction between expected post-sparring soreness and evolving pathology [29].

Finally, the field would benefit from increased implementation science. Evidence is only useful if it changes practice. Studies should therefore evaluate whether surveillance systems are acceptable to coaches, whether weight management policies are enforceable, whether recovery interventions can be delivered in real training environments, and whether athlete education changes help-seeking behavior. Open protocols, shared data dictionaries, and multicenter collaboration would substantially increase comparability and cumulative knowledge.

## 5. Conclusions

The articles in this Special Issue collectively show that sports medicine for combat sports is expanding from descriptive injury documentation toward a broader model of athlete health and safer performance. The included studies and review address injury burden, monitoring, recovery, weight-cutting risk, and emergency recognition across karate, MMA, and kickboxing. Their combined message is that athlete protection in combat sports requires more than treating injuries after they occur: athlete protection requires prospective surveillance, contextualized monitoring, tested interventions, safer weight-management cultures, emergency vigilance, and practical implementation in gyms and competitions. The next stage of research should be collaborative, longitudinal, and policy-aware, while remaining sensitive to the technical and cultural realities that make combat sports unique.

## Figures and Tables

**Table 1:** Main themes represented in this Special Issue.

Special Issue Article	Primary Contribution	Future Direction Highlighted
de Wet and Yelverton [17]	Descriptive injury profile in South African Shotokan karate, including injury prevalence, common injury types, and tendency to continue training while injured.	Prospective, medically verified, and exposure-normalized surveillance in training and competition.
Muracki et al. [18]	Comparative body composition and phase angle data in MMA and powerlifting athletes.	Longitudinal monitoring that links body composition with load, hydration, recovery, weight-cycle timing, injury, and performance.
Trybulski et al. [19]	Randomized trial testing sports massage and blood flow restriction/cooling recovery strategies in trained MMA athletes.	Larger pragmatic trials with active comparators, longer follow-up, mechanistic markers, and implementation outcomes.
Kużdżał et al. [20]	Scoping review of weight-cutting effects on sleep, recovery, and injury outcomes in combat sports.	Policy-relevant studies of safer weight management strategies, including women, youth, and recreational athletes.
Beca et al. [21]	Case report of acute compartment syndrome after repeated calf kicks in elite kickboxing.	Education and clinical pathways for early recognition of rare but severe sport-specific complications.

## Abbreviation

The following abbreviation is used in this manuscript:

MMA	Mixed Martial Arts
